# Marijuana Use among African American Older Adults in Economically Challenged Areas of South Los Angeles

**DOI:** 10.3390/brainsci9070166

**Published:** 2019-07-16

**Authors:** Sharon Cobb, Mohsen Bazargan, James Smith, Homero E. del Pino, Kimberly Dorrah, Shervin Assari

**Affiliations:** 1 School of Nursing, Charles R Drew University of Medicine and Science, Los Angeles, CA 90059, USA; 2 Department of Family Medicine, College of Medicine, Charles R Drew University of Medicine and Science, Los Angeles, CA 90059, USA; 3 Department of Public Health, Charles R Drew University of Medicine and Science, Los Angeles, CA 90059, USA; 4 Department of Family Medicine, University of California, Los Angeles (UCLA), Los Angeles, CA 90095, USA; 5 Department of Psychiatry and Human Behavior, College of Medicine, Charles R Drew University of Medicine and Science, Los Angeles, CA 90059, USA; 6 Department of Psychiatry and Biobehavioral Sciences, University of California, Los Angeles (UCLA), Los Angeles, CA 90095, USA

**Keywords:** African American, black, older adult, marijuana use

## Abstract

**Purpose:** This study explored demographic, social, behavioral, and health factors associated with current marijuana use (MU) among African American older adults who were residing in economically challenged areas of south Los Angeles. **Methods:** This community-based study recruited a consecutive sample of African American older adults (*n* = 340), age ≥ 55 years, residing in economically challenged areas of South Los Angeles. Interviews were conducted to collect data. Demographics (age and gender), socioeconomic status (educational attainment, income, and financial strain), marital status, living alone, health behaviors (alcohol drinking and cigarette smoking), health status (number of chronic medical conditions, body mass index, depression, and chronic pain), and current MU were collected. Logistic regression was used to analyze the data. **Results:** Thirty (9.1%) participants reported current MU. Age, educational attainment, chronic medical conditions, and obesity were negatively associated with current MU. Gender, income, financial strain, living alone, marital status, smoking cigarettes, drinking alcohol, depression, and pain did not correlate with MU. **Conclusion:** Current MU is more common in younger, healthier, less obese, less educated African American older adults. It does not seem that African American older adults use marijuana for the self-medication of chronic disease, pain, or depression. For African American older adults, MU also does not co-occur with cigarette smoking and alcohol drinking. These results may help clinicians who provide services for older African Americans in economically challenged urban areas.

## 1. Background

Very little epidemiological information exists on marijuana use (MU) among African American older adults, specifically those who live in economically challenged urban settings [1]. This information is essential for the design and implementation of cessation and treatment programs for African American older adults in such settings [2]. The patterns and predictors of MU in the African American community differ from those of other communities [3,4]. Economically challenged African American communities may have high availability of marijuana, marijuana initiation, and use, combined with poor access to cessation programs. Low access to MU cessation programs may operate as a vulnerability factor for this population, which increases the risk of substance use problems [5,6,7,8,9,10]. Telescoping effect is a phenomenon that describes the more rapid transition of marginalized African Americans from substance use to poor outcomes. Such a phenomenon explains why African American communities are at an increased risk of risky trajectories of substance use [5,6,7,8,9,10]. As a result, although African Americans show a lower prevalence of substance use, they are more likely to develop undesired substance use outcomes [5,6,7,8,9,10]. 

Demographic factors [11] such as age, period, cohort effects, and gender all influence MU [12,13,14,15,16,17,18]. Among adults, age increases the risk of lifetime substance use; however, current use is more common at younger ages and in more recent cohorts [12]. Gender is a main determinant of substance use—across studies, males are more likely to use tobacco, alcohol, and marijuana [19]. 

Low socioeconomic status (SES) reduces population health [20] and increases the risk of use of a wide range of substances such as tobacco [21,22], alcohol [23], and marijuana [24,25]. Low SES is also a major driver of racial and ethnic health disparities that explain the worse behavioral and health outcomes in the African American community, relative to Whites [26]. SES indicators, such as educational attainment, financial strain, and income, impact health [20] and health behaviors such as MU [24,25]. Some recent literature, however, suggests that the health effects of SES are smaller for African Americans than Whites [20]. For example, as a result of practices and preferences of the labor market which marginalizes highly educated African Americans, highly educated African Americans are less likely to secure high paying jobs than Whites. This phenomenon reduces the central role of education level on health and behaviors of African Americans [27]. As a result, education attainment may generate less health for African Americans than Whites [21,27], also known as minorities diminished returns (MDRs) [21,27,28]. As a result, education attainment may have smaller effects for African American individuals [21,27] than for Whites. While education attainment, income [22], and financial strain [29] shape health and health behaviors of populations, there is a need to test how these SES indicators impact the MU of African American older adults. 

Use of tobacco and alcohol may be associated with MU [30]. This is in part because the use of various substances has shared risk factors [31]. One mechanism for the association between tobacco, alcohol, and marijuana use is that some of these substances may operate as a gateway to the other substances [3]. For example, individuals who currently use alcohol or tobacco are more likely to use marijuana in the future [30]. 

Health problems may also covary with MU. First, there is large body of research suggesting a negative association between obesity and MU [32]. This literature has proposed multiple mechanisms, including that drugs and food may be two non-overlapping reward pathways [32]. Thus, people may have great interest in either food or drugs, but not both, to cope with stress [33]. Second, people may also turn to MU to cope with pain [13] or depression [14]. In this case, we expect higher risk of MU in individuals who have high levels of depressive symptoms [14] or pain [13]. 

Most of the literature on MU among African Americans is on younger age groups [11,14]. As a result, we have limited knowledge about how demographics, socioeconomics, health behaviors, and health correlate with MU among African American older adults [15]. There is also a need to expand the existing literature on demographic, social, behavioral, and health determinants of MU in African American older adults who reside in economically challenged urban areas. This is particularly important given the transition in the patterns of MU following the legalization of marijuana. Similarly, medical marijuana may be used by individuals, particularly older adults, and this effect may depend on whether recreational or medical marijuana is legal or not [13].

### Aims

The current study explored demographic, social, behavioral, and health determinants of current MU in economically challenged African American older adults. We hypothesized that MU is more common in people who are younger, fit, healthy, male, have low educational attainment, have high levels of financial strain, are those who smoke and drink, and have depression and pain. 

## 2. Methods

### 2.1. Design and Setting

A survey was performed in economically challenged areas of south Los Angeles between 2015 and 2018 [16,17,18,34]. The survey included a structured face-to-face interview which collected data on demographic factors, SES, health behaviors, health status, and MU. Participants were living in the Service Planning Area 6 (SPA 6), Los Angeles County, California. SPA 6 is one of the most economically challenged urban areas, with 58% of adults having income levels less than 200% of the federal poverty line (FPL) and 36% of the population being uninsured [35,36]. 49% of older adults residing in SPA 6 are African Americans. Between 2013 and 2015, the percentage of homeless AA individuals in SPA 6 rose from 39% to 70%.

### 2.2. Participants and Sampling

A non-random sample of African American older adults was recruited for this study. Participants were sampled from predominantly African American housing units and senior centers that were located in south Los Angeles. Participants were 340 African Americans. Individuals were eligible if they were (a) African American/Black, (b) non-institutionalized, (c) aged 55 years or older, and (d) lived in south Los Angeles (LA). Exclusion criteria were (a) enrollment in skilled nursing facilities, (b) current enrollment in a clinical trial (because the intervention can interfere with MU and other health behaviors), and (c) severe cognitive deficit (not being able to consent and conduct the interview). 

### 2.3. Institutional Review Board (IRB)

The Charles R. Drew University of Medicine and Science (CDU) IRB approved the study protocol. All respondents signed a written informed consent.

### 2.4. Study Measures

#### 2.4.1. Independent Variables

*Socio-economic status (SES):* Three SES indicators were included. Educational attainment, financial strain, and income. Education attainment was conceptualized as years of schooling. This variable was treated as an interval variable. Financial strain was measured using three items borrowed from the Pearlin’s list of financial difficulties that are commonly experienced by low SES people [37]. These items cover not having enough money for essential needs such as food, clothes, rent/mortgage, and utility bills. Responses were on a Likert scale ranging from 1 ‘never’ to 5 ‘always’. We calculated a total score a score that reflected overall financial difficulties. (Cronbach’s alpha = 0.923). Household monthly income was a continuous measure (in USD $1000).

*Demographic Characteristics:* Age (years) and gender (male, female) were measured. Age was a continuous variable. Gender was a dichotomous measure. 

*Living Arrangements*: A dichotomous variable reflected participants’ living arrangement. Participants’ living arrangement was measured using a single item. The variables were 1 (living alone) and 0 (there are any other members accompanying them) [38]. 

*Marital Status*: Participants’ family type (marital status) was measured using a single item self-report. This variable was treated as a dichotomous variable: married = 1, non-married = 0.

*Obesity:* Obesity was measured by measurement of weight and height. Height and weight were measured in inches and pounds, respectively. Then, height and weight were converted to meters and kilograms. Body mass index (BMI) was then defined as weight (kilograms) divided by height (meters) squared. 

*Number of Chronic Medical Conditions (CMC):* Individuals were asked if they were ever told that they had the following chronic medical conditions: hypertension (HTN), heart disease, stroke, cancer, diabetes (MD), thyroid disease, chronic obstructive pulmonary disease (COPD), asthma, osteoarthritis, gastrointestinal (GI) disease, rheumatoid arthritis (RA), and lipid disorder. Self-reported CMCs were valid and reliable [39]. We calculated the total number of CMCs as reported by the individual. 

*Depression:* This study measured depression using the 15-item Geriatric Depression Scale (Short Form) (GDS-SF) [40]. Results range from 1 to 15, with a higher score indicative of severe depressive symptoms. The GDS-SF has shown very good reliability and validity, and has been commonly used in community and clinical settings [40].

*Pain:* Pain intensity was measured by the McGill Pain Questionnaire (Short Form 2) (MPQ-SF-2) [41]. This scale has 22 pain items asking about the experience of various types of pain in the past week. Each item was on an 11-point rating scale ranging from 0 (none) to 10 (worst possible). A total pain score was calculated. A higher score reflected more intense chronic pain [41]. 

*Tobacco Use:* Participants were asked whether they smoke cigarettes. The exact question was: “How would you describe your cigarette smoking habits?” Response items included never smoked, previously smoked, and current smoker. We defined a dichotomous variable as current smoker versus other statuses. 

*Drinking Alcohol:* Participants’ alcohol use was asked using this question: “Do you drink alcohol?” The response items included *yes* and *no*. Drinking alcohol was a dichotomous variable.

#### 2.4.2. Outcome Variables

*Current Marijuana Use (MU):* Two items were used to measure current use of marijuana: (1) *“Are you taking marijuana or any related products for pain?”*, and (2) *“In the past year have you been treated by Compassion provider for marijuana related products?”* [42].

### 2.5. Statistical Analysis

We used SPSS 23.0 (IBM, New York, NY, USA) for data analysis. To describe the sample, we reported frequencies (*n*) and relative frequencies (%) of the categorical variables. We calculated the average number of CMCs for the analysis. Means and standard deviations (SD) were reported for continuous measures. We used the non-parametric Spearman correlation test (zero order correlation) to estimate the bivariate correlations between the study variables. We applied logistic regression models with MU as the outcome (the dependent variable) and demographics, SES, health behaviors, and health as the predictors (independent variables). As almost all participants had some type of health care coverage, we did not include health insurance to our logistic regression models. We reported the odds ratio (OR), and their associated standard error (SE), 95% confidence intervals (95% CI), and *p* values from our logistic regression models. 

## 3. Results

### 3.1. Descriptive Statistics

Table 1 describes the study variables. All participants were at least 55 years old. Participants had an average age of 69.6 (SD = 9.3) years old. From all our participants, 63.2% were female. From our participants, 9.1% (*n* = 30) reported MU.

### 3.2. Bivariate Analysis

Table 2 shows a summary of the bivariate correlations between the study variables, using a non-parametric correlation Spearman test. Age, education attainment, obesity, and number of CMCs were negatively correlated with current MU, however, other variables, such as financial strain, depression, and pain, were not associated with MU. Smoking cigarettes and drinking alcohol also did not correlate with current MU.

### 3.3. Multivariable Analysis

Table 3 shows the results of a logistic regression model with current MU as the outcome. According to this model, age, educational attainment, chronic medical conditions, and obesity were negatively associated with current MU. Gender, monthly household income, financial strain, living alone, marital status, smoking cigarettes, drinking alcohol, depression, and pain did not predict current MU (Table 3).

## 4. Discussion

Age, educational attainment, obesity, and CMCs were associated with current MU in our sample of African American older adults in economically challenged areas of south Los Angeles. While lower age was correlated with current MU, gender was not linked to the same behavior. Educational attainment, but not income or financial strain, was associated with MU. Neither cigarette nor alcohol use was associated with current MU in this population. However, current MU was associated with a lower risk of obesity, and individuals who reported MU were less likely to have CMCs. Our findings were in line with the research that shows a negative association between BMI and current MU [43], although not all studies have shown such negative associations [44].

Prevalence of MU was very low in this study, which is slightly higher than the national prevalence rate of 2.9% among older adults [15]. Currently, white women are identified as the most prevalent users of marijuana among older adults [45]. However, the results should also be interpreted in the full context of marijuana use in the whole age range of the African American population. MU use is higher among African American young adults compared to age-matched Whites, as they are more likely to use marijuana before tobacco [46]. However, this may not translate to older African Americans, who may be less knowledgeable about MU-associated health risks. In addition, MU is heavily stigmatized, and individuals may be reluctant to report usage, which may be a contributing factor to the reported low rate of MU among this population [47]. MU has been heavily criminalized for decades within the African American population, with high rates of marijuana-related arrests and negative interactions with the criminal justice system for use and possession [48]. Even though there is a national wave of legalizing medical and/or recreational marijuana in multiple states—including California—these African American older adults may still not want to report MU because of fear and stigma. The results should also be interpreted with the knowledge regarding the types of consumption methods of MU, which may include marijuana in cigarillos wraps, commonly known as “blunts”, that are prevalent in stores in economically challenged areas and physically more harmful. This presents a striking difference, as Whites are more likely to use other methods of marijuana consumption, such as edibles, which are more costly and harder to access for this sample population [49]. Finally, we cannot rule out the likelihood of measurement bias, and should use multiple items to measure MU. Thus, there is a need for more research on this topic. 

We did not find any linkage between MU and depression or pain, suggesting that African American older adults in economically challenged areas of Los Angeles do not use marijuana to self-medicate their depression and pain. Although this is a plausible explanation for our findings, other explanations should also be considered. Among African Americans, early-starting MU is linked to depressive symptoms and/or depressive disorders with underlying adverse child experiences [50,51]. Yet, this relationship has been primarily explored among adolescent and young African American adults. As there was a low rate of depression and pain among African American older adults in this sample, use of marijuana for depression and pain cannot be accurately assessed in this study. More research is needed on the motivations behind MU in African American older adults.

The profile of the typical marijuana user is younger, less educated, healthier (less CMCs), and more physically fit (less obese). MU among African American older adults does not co-occur with smoking cigarettes and drinking alcohol, which differs from the typical pattern of an older adult marijuana user [2]. Instead, MU may be a sporadic or inconsistent behavior, rather than consistent with other health risk behaviors, particularly substance use. Still, understanding the profile of marijuana users in African American older adults may help the delivery of health services and increase health education [52]. 

While educational attainment was associated with MU, we did not find protective effects of income and low financial strain on MU. The lack of association between MU and other SES indicators could be due to MDRs; hence, income may not be particularly protective against risk behaviors such as substance use [21,27,28].

More efforts should be geared toward the health education of African American older adults, who may not be aware of the health implications of marijuana. There is an increase in the number of policies, both at the local and state levels, addressing medical and recreational MU. There is a need for health systems, including providers and various community organizations, to provide knowledge about marijuana to the African American community. Our findings showed that adults likely to use marijuana are younger and have fewer CMCs. Yet, chronic MU may lead to future health issues as individuals age. Further research should focus on the health profile of African American older adults with MU. 

### Limitations

The study is not without methodological and conceptual limitations. First, its cross-sectional design limits any causal inferences. Second, the non-random sample limits the generalizability of the results to the broader African American community. Third, the study used a simplistic measure of current MU. As a result, this study was unable to assess either past or lifetime use of marijuana. Frequency of MU, types of MU consumption, and access and availability of MU were also not obtained. In addition, we cannot rule out the possibility of measurement bias, as we relied on self-reported marijuana use. Still, because of the stigma and sensitivity of marijuana, especially within this population, self-reported MU may be the best method to obtain information. In addition, data collection occurred prior to California legalizing recreational marijuana. Therefore, marijuana users within this sample may have not reported use because it may have been potentially illegal. These are important factors to consider when examining the relatively low prevalence of marijuana use in this population. Another limitation of the study is the lack of epidemic data from younger African Americans for comparison. If such data were available, the analysis and arguments could be significantly improved. Yet, there is a need to consider generational patterns when examining the two groups, as attitudes toward MU have become more positive among younger populations, which may be linked to higher prevalence among younger groups. Fourth, the study only included older African Americans who were residing in low income inner cities. Even though this decreased generalizability, few studies have focused on substance use among African American older adults residing in underserved areas, especially with MU. This is an important study to assess the prevalence and significant factors to start to understand the profile of African American older adults with MU. Fifth, our measure of CMCs was not exclusive. Other conditions, such as respiratory diseases, neurological diseases, and other diseases impacted by chronic MU could be included in future studies. The findings thus may differ for any other groups, even African Americans who have higher SES or those who are biracial or multi-racial. 

The results reported here should be regarded as preliminary. More research is needed with more detailed information on MU. Despite these limitations, this study contributes to our knowledge of MU use in a population (older African American adults) about which little is currently known.

## 5. Conclusions

Educational attainment seems to protect African American older adults against current MU. At the same time, current MU is more common in younger, healthier, and more physically fit older African American adults. For African American older adults in poor urban areas, current MU may not co-occur with the use of tobacco and alcohol. Further studies should focus on assessing various aspects of MU, such as lifetime use, accessibility, and consumption of marijuana-related products, such edibles, among these adults. We did not find strong evidence suggesting that African American older adults turn to MU for self-medication of pain and depression.

## Figures and Tables

**Table 1:** Descriptive statistics (*n* = 340).

Characteristics		
	**Mean**	**SD**
Age (years)	69.60	9.33
Educational attainment (years)	12.73	2.13
Financial strain	12.10	6.15
Monthly household income (USD $1000)	2.69	1.10
Chronic medical conditions (CMC)	4.35	1.83
Depressive symptoms	3.36	3.04
Chronic pain	2.51	2.44
	***n***	***%***
Gender		
Men	125	36.8
Women	215	63.2
Cigarette smoking (current)		
No	107	31.7
Yes	231	68.3
Alcohol drinking		
No	190	56.0
Yes	149	44.0
Obesity		
No	188	55.3
Yes	151	44.4
Current marijuana use		
No	309	90.9
Yes	31	9.1

**Table 2:** Bivariate correlations.

	1	2	3	4	5	6	7	8	9	10	11	12
1 Gender (female)	1	0.12 *	0.10	−0.14 *	−0.09	0.13 *	−0.17 **	0.15 **	0.23 **	−0.05	0.06	−0.12 *
2 Age		1	−0.19 **	−0.29 **	0.04	0.43 **	−0.34 **	0.07	−0.07	−0.24 **	−0.22 **	−0.11 *
3 Educational attainment (years)			1	−0.09	0.22 **	−0.06	0.06	−0.10	0.01	−0.15 **	−0.03	−0.07
4 Financial strain				1	−0.15 **	−0.16 **	0.14 *	0.13 *	0.04	0.44 **	0.37 **	0.03
5 Monthly household income (USD $1000)					1	0.07	−0.05	−0.16 **	0.10	−0.13 *	−0.18 **	0.02
6 Smoking						1	−0.33 **	0.04	0.19 **	−0.16 **	−0.16 **	−0.07
7 Drinking							1	0.04	−0.05	0.05	0.17 **	0.07
8 Chronic medical conditions (CMCs)								1	0.12 *	0.25 **	0.42 **	−0.17 **
9 Obesity									1	0.09	0.09	−0.12 *
10 Depression										1	0.44 **	−0.04
11 Chronic pain											1	−0.02
12 Current marijuana use												1

*, *p* < 0.05; **, *p* < 0.01.

**Table 3:** Summary of multivariable logistic regression models with marijuana use (MU) as the outcomes. OR = odds ratio, CI = confidence intervals.

	OR	95% CI	*p*
Gender (female)	0.73	0.32–1.67	0.45
Age	0.94	0.89–1.00	0.04
Educational attainment (years)	0.81	0.68–0.96	0.02
Financial strain	1.01	0.94–1.08	0.78
Monthly household income (USD $1000)	1.10	0.77–1.58	0.60
Smoking	1.27	0.49–3.28	0.62
Drinking	1.22	0.51–2.90	0.65
Chronic medical conditions (CMC)	0.69	0.52–0.91	0.01
Obesity	0.42	0.17–1.04	0.05
Depression	0.92	0.77–1.09	0.32
Chronic pain	1.13	0.92–1.39	0.24
Constant	276.52		0.03
